# Hospital preparedness for major incidents in Sweden: a national survey with focus on mass casualty incidents

**DOI:** 10.1007/s00068-022-02170-z

**Published:** 2022-12-08

**Authors:** Louise Söderin, Joakim Agri, Elsa Hammarberg, Kristina Lennquist-Montán, Carl Montán

**Affiliations:** 1 Department of Molecular Medicine and Surgery, Vascular Surgery, Karolinska Institutet, Stockholm, Sweden; 2 Department of Global Public Health, Karolinska Institutet, Stockholm, Sweden

**Keywords:** Mass casualty incident, Disaster planning, Disaster medicine, Surge capacity, Hospital preparedness, Sweden

## Abstract

**Introduction:**

Mass-casualty incidents, MCI, pose a constant threat on societies all over the world. It is essential that hospital organizations systematically prepare for such situations. A method for repeated follow-up and evaluation of hospital disaster planning is much needed.

**Aims:**

To evaluate Swedish hospitals´ disaster preparedness with focus on MCI through a web-based survey to highlight areas in need of improvement to ensure better preparedness and resilience.

**Materials and methods:**

An online survey was sent to all Swedish emergency hospitals (*n* = 87, 49 emergency hospitals). One respondent per hospital answered questions about the hospital’s disaster planning, training, key functions, and preparedness. The survey was developed based on current knowledge on key areas of interest for all-hazard preparedness, including the WHO’s guidelines. The survey was open between September 6th and November 1st, 2021.

**Results:**

39 hospitals (34 emergency hospitals) from 18/21 regions participated. Main findings included marked differences between regions and hospital types regarding contingency plans, organization, formal education for key functions, disaster training and triage systems.

**Conclusions:**

Generally, Swedish hospitals cover most key areas in disaster preparedness, but no hospital appears to have a full all-hazards coverage, which leaves room for improvement. There are large variations between the different hospitals’ preparedness, which need to decrease. Several hospitals expressed a need of national guidelines for developing equivalent contingency plans. The study-method could be used for monitoring compliance with current laws and guidelines.

## Introduction

Mass casualty incidents (MCI) and other major incidents (MI) occur repeatedly and demand special healthcare system preparedness. Worldwide, there are global trends of increased MCIs following terrorism, changing security situation and natural disasters [1]. To effectively evaluate preparedness before an MI occurs is essential to achieve optimal resilience. Simulation exercises and repeated training are important factors for success.

**Fig. 1:**
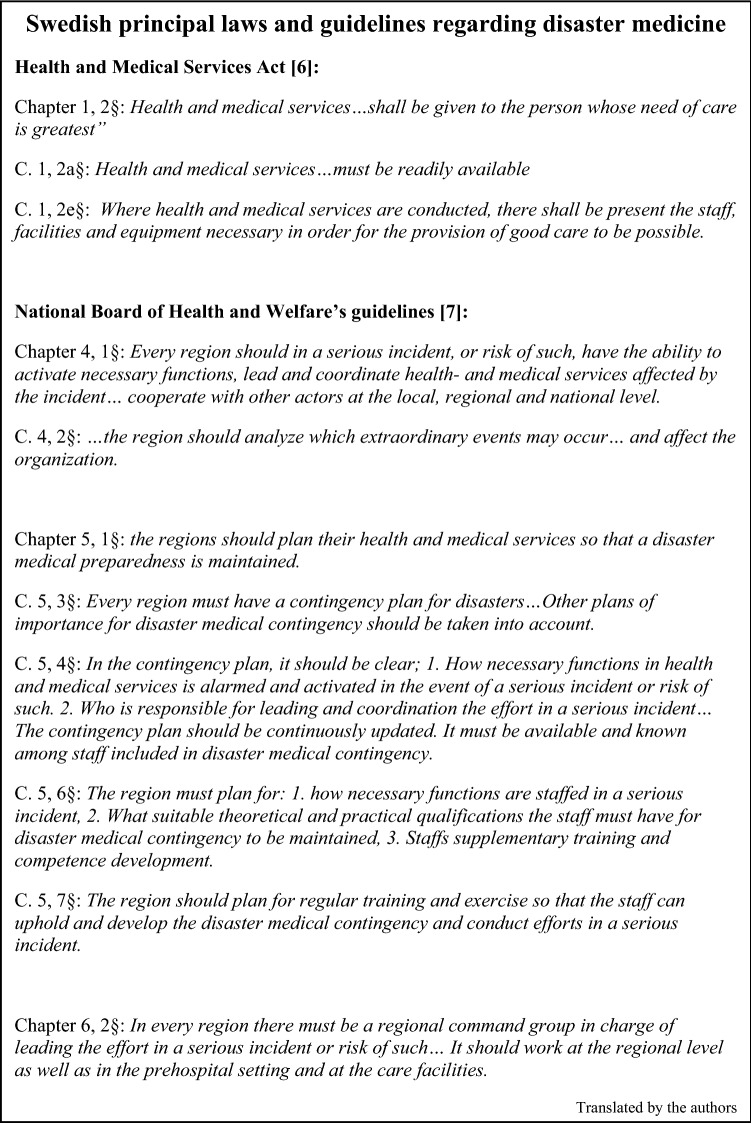
Swedish laws and guidelines on disaster medicine, main paragraphs. Freely translated

Swedish healthcare has historically been pioneers in disaster medicine, but in recent MIs, insufficient preparedness and need of improvement has been seen [2–4]. In the past 15 years, political decisions have resulted in reduced resources for preparedness [5]. Recent global challenges such as the pandemic, terrorism and the invasion of Ukraine has increased focus on these issues, and initiatives to improve preparedness has gained urgency. In Sweden, the Health and Medical Services Act (HSL) [6] and the National Board of Health and Welfare (NBHW) regulate preparedness work through guidelines and legislation [7]. For main laws and guidelines, see Fig. 1.

An investigation after the terror attack in Stockholm 2017 stated that the medical effort generally worked well but emphasizes great needs for review of contingency plans, communication systems and routines for calling in staff [8]. These factors are some of the cornerstones for disaster preparedness according to previous studies [1, 8–10]. The WHO's checklist for all-hazards approach in disaster preparedness includes, among others, surge capacity, triage and supply management [9–11]. Contingency plans including different types of disasters, such as epidemics and network disruptions, are recommended [11, 12]. It is also well-established that well-functioning contingency plans must be accessible, concise and well known amongst concerned staff [1, 8].

Each of Sweden’s 21 healthcare regions should have a regional contingency plan, and each hospital a local contingency plan, according to the HSL and the NBHW’s guidelines [6, 7, 15, 16]. How the plans are organized, practiced and known amongst staff is not known. A systematic method for regular control and follow-up on how hospital preparedness regulations and guidelines are implemented and followed is needed.

### Aims

To identify areas in need of improvement in preparedness for incidents and disasters at Swedish emergency hospitals, with focus on mass casualty incidents, using a web-based survey, and to investigate how well current legislation and guidelines on preparedness are complied with.

## Materials and methods

### Study design

A descriptive cross-sectional study was conducted through a web-based survey. 87 hospitals were invited to participate with primary focus on participation from the 49 Swedish emergency hospitals (i.e. hospitals with a full-scale emergency department receiving both medical and trauma patients) [13]. Non-emergency hospitals (hospitals with an emergency department with for instance limited opening hours, only for medical patients, etc.) can also be involved in MIs, especially in the many rural parts of Sweden where distance to an emergency hospital is large. It was, therefore, of interest to investigate preparedness on some of these.

The survey (appendix 1) was designed based on aspects identified to be of greatest importance for adequate preparedness, including the WHO's checklist [12]. It consisted of 67 questions formulated in general terms covering following sections:

- Background information
- Contingency plans
- Major incident and disaster exercises
- Hospital Command Group, communication and triage
- Surge capacity and regional coordination
- Real-life events
- Supply management, mobilization at the hospital, review of key areas

The survey was reviewed and deemed suitable for the aim with regards to content by Sten Lennquist, professor emeritus in Disaster Medicine. Due to confidentiality reasons, several surveyed hospitals did not wish to provide a copy of the contingency plan and comparison between provided answers and actual contingency plan was therefore not conducted.

The survey was conducted through a software program well suited for the purpose and was open between September 6th and November 1st, 2021. Respondents could pause the survey and, if so whished, choose not to disclose information requested. An initial reminder was sent after 3.5 weeks, after which weekly reminders were sent until last response date (total 4 reminders).

### Data analysis

Analysed data were mainly descriptive and extracted to Microsoft Excel (version 16.43, 2020) for analysis. For descriptive data, averages, medians and percentages were used. For continuous data, standard deviation was calculated. This was performed both for collected data and divided according to hospital types. Qualitative data were analysed through content analysis where main themes for each question were identified.

### Ethical considerations

The nature of the study entails a certain risk of accumulation of potentially sensitive data that could constitute a security threat to individual hospitals, regions or Swedish healthcare in general. To reduce the risk, questions asked were kept at a general level, minimizing the amount of sensitive data. Data were handled with confidentiality according to regulations at Karolinska Institute. Results were reported so that individual hospitals or regions were not disclosed. The benefit of an overall study of Swedish disaster preparedness was assessed greater than potential security risks. As the study was conducted at the organizational level, there is no risk to autonomy or integrity of individuals. The study was approved by the Swedish Ethical Review Authority, no. 2021–02865.

## Results

39 out of a total of 87 invited hospitals answered the survey (45%). 34 of responding hospitals were from the 49 emergency hospitals, giving a response rate of 69.4% among these hospitals. The other five were non-emergency hospitals that either had an emergency room with limited opening hours or a 24-h local emergency unit. However, some of the respondents indicated that more than one hospital was part in the contingency organization they represented, leading to a higher actual number of respondents than the factual one. For simplicity and security in the statistics, the numbers presented below are 39 answers of the survey that has been provided, although the results can be representative for more hospitals than the 39 answered surveys. 7 hospitals chose not to participate due to confidentiality. 18/21 (86%) regions were represented, with a good geographical spread. All hospital types were represented; university hospitals in the form of regional trauma centres (RTC), non-trauma centre university hospitals (NTCUH), and smaller hospitals including county hospitals (CH), county district hospitals (CDH) and local hospitals (LH) (Table 1). The majority had a trauma admission area of 100,000–500,000 people. The five non-emergency hospitals were LH or CDH.

**Table 1:** Number of respondents per hospital type

Hospital type		*n*		Percent (%)	
University Hospitals, UH	Regional Trauma Centres, RTC	6	4	15.4	10.3
	Non-Trauma Centre University Hospitals, NTCUH		2		5.1
County Hospitals, CH		16		41	
County District Hospitals, CDH		15		38.5	
Local Hospitals, LH		2		5.1	

Some hospitals provided one answer for a total of up to three hospitals making up the contingency organization, meaning that the number of participating hospitals can be considered to be higher than the number of respondents. *n* = 39

A clear majority (34/39) of the hospitals’ respondents were contingency coordinators (CC). One response per hospital was requested. Three hospitals answered twice, in which cases only the response from the CC was analysed as the survey was mainly aimed at CCs. Results are presented by the surveys main areas.

### Information about the hospital and key coordinating functions

**Fig. 2:**
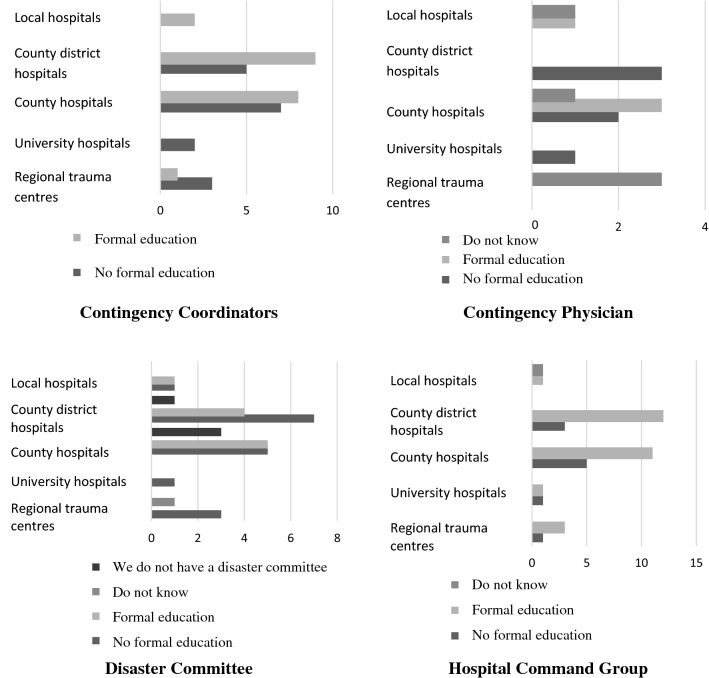
Requirements for formal education in disaster medicine for hospital key functions. The figure regards to the questions “What type of education in disaster medicine does the hospitals contingency coordinator/physician have?” and “Do you have any requirements for a formal education in disaster medicine for those in your disaster committee/Hospital Command Group?”. *n* = 39

All but one hospital had a nurse as CC. Two hospitals (1 CH, 1 CDH) had no function responsible for disaster preparedness. At UH, the CC in 4/6 hospitals (67%) had basic nurse training. At CH and CDH, 15/31 of CCs were specialist nurses, most commonly specialized in anaesthesia (*n* = 8), intensive care (*n* = 2), or emergency/prehospital care (*n* = 6). Regarding the CCs formal training in disaster medicine, higher levels were seen at smaller hospitals (CH, CDH, LH) compared with all UH. Only one UH (17%) had a CC with formal education, whereas for CH it was 8/15 (53%), CDH 9/14 (64%) and LH 2/2 (100%) (Fig. 2).

4/6 (67%) of UH had a contingency physician. In smaller hospitals, such function was significantly less common; 6/16 (37%) and 3/15 (20%) respectively at CH and CDH. The proportion requiring formal training in disaster medicine for contingency physicians was higher at CH and CDH compared with UH (3/4 (75%) of RTCs "do not know", 1/4 (25%) stated "no formal education". 3/6 (50%) of CH, 3/3 (100%) of CDH had educational requirements (Fig. 2)).

A “Disaster Committee” for preparedness was present at all UH and most of CH and CDH (21/31, 68%). No UH required formal training to join the committee while 5/10 (50%) CH and 4/11 (36%) CDH required this (Fig. 2). One CH did not have either a contingency coordinator, contingency physician or disaster committee.

### Contingency plans

All hospitals except one CDH had a hospital-wide contingency plan. In addition, 5/6 (83%) UH had synchronized department-specific plans, for CH and CDH 8/16 (50%) and 5/15 (33%) had this. Great variation in plan length was seen (median 34 pages, min–max; 13–80). LH generally had shorter contingency plans (median 17.5). The majority updated their contingency plan in the past two years, except 5 CH and 6 CDH. All but three had updated the MCI plan 2018 and onwards. Among those who had updated all parts of the plan, CH and CDH dominated; 11/16 (69%) and 11/15 (73%) respectively compared with UH (1/6, 17%).

Large differences were seen in described structure of contingency plans, complicating structure analysis. 11/31 CH and CDH only use the regional contingency plan, in 7/11 cases with local action cards. Regarding which employees read the contingency plan, an even distribution was seen between "new employees", "up to each employee" and "according to guideline". Seven hospitals wrote that the responsibility had been delegated to the units and four hospitals that “The guideline is that all new employees should read it. This is probably not the case”.

Regarding separate plans for different types of MIs, most notably was, that not all hospitals had a plan for network disruptions. All participating UH, but only 13/16 (81%) of CH and 14/15 (93%) of CDH had this. In general, UH used action cards for more different functions. One CH did not use action cards at all.

### Major incident and disaster exercises

One CH and two CDH stated that they never conduct disaster drills. Among others, the just over half of the respondents said either that all trauma-receiving employees or a few per year according to a rolling schedule participate in disaster drills. Five hospitals stated that only HCG practices. The 26/39 (67%) practice different scenarios. One CDH did not evaluate exercises. Most evaluate either through review in the disaster committee (8/39, 20%) or through evaluation of staff knowledge development (11/39, 28%). A clear majority (30/39, 77%) used the results to update and improve contingency plans.

### Hospital Command Group

All UH and 25/31 (81%) of CH and CDH had a multidisciplinary and trained HCG. At four CH and CDH, HCG consisted of regular managers or regional management. The included functions were fairly even at different hospitals, apart from nursing professions, where no RTC had such representation (for others; 33–100%, 50% median). Three CH/CDH stated that it varied depending on the incident. HCG at all but one CH and one CDH worked according to a special structure, most often the NATO model for staff methodology. All respondents said that HCG is trained, 20/39 (51%) trained MCI 2019. Regarding requirements for training in disaster medicine, 4/6 (67%) of UH, 11/16 (69%) of CH, 12/15 (80%) CDH and 2/2 (100%) of LH had such requirements (Fig. 2).

### Communication

Regarding disaster communication systems, the 21/39 (54%) planned to use regular systems with pagers, mobile phones and landlines, in several cases with addition of radio communication. 3/6 (50%) of UH, 9/16 (56%) of CH, 8/15 (53%) of CDH and 2/2 (100%) of LH had a tested backup system for communication. One CH had no backup system at all. 30/39 (77%) had a designated person responsible for updating hospital staff contact lists. 2/6 (33%) of UH, 3/16 (19%) of CH and 1/15 (7%) of CDH had no system to ensure updated staff contact lists.

### Triage

All hospitals had a plan for where primary triage in an MI would take place. For secondary triage, one quarter stated that trauma teams decide where it would take place, the rest had specified the location in advance. Regarding triage method, large variations were seen (Table 2). Two hospitals responded that they did not know which method was used.

**Table 2:** Triage methods in the event of a disaster

	MIMMS (*n*)	RETTS (*n*)	Triage Sieve (*n*)		Other (*n*)
Which triage system will be used for primary triage in the event of an MCI/disaster?					
RTC	0	0	0		4
NTCUH	0	1	1		0
CH	2	3	6		5
CDH	1	4	3		7
LH	0	0	0		2
Which triage system will be used for secondary triage in the event of an MCI/ disaster?					
RTC	0	1		3	
NTCUH	0	2		0	
CH	1	11		4	
CDH	1	7		7	
LH	0	1		1	

The table shows triage methods described in text answers that are planned to be used in the event of a disaster, divided according to the most common answers. Within the group "other", very varied answers were seen, including "do not know", "not relevant" and "regular triage method"

*RTC* regional trauma center, *NTCUH* non-trauma center university hospital, *CH* county hospital, *CDH* county district hospital, *LH* local hospital, *RETTS* Rapid Emergency Triage and Treatment System, *MIMMS* Major Incident Medical Management and Support. *n* = 39

### Surge capacity and regional coordination

3/4 (75%) RTC had evaluated the hospital surge capacity. For CH, this was 6/16 (38%) and for CDH 3/15 (20%), both NTCUH replied "do not know". No surge capacity evaluation had been done at 7/16 (44%) of CH and 9/15 (60%) of CDH. Two CH described difficulties evaluating capacity due to large variations in available resources, one of which said that capacity revaluation is done continuously due to "significantly reduced margins". Three hospitals (2 RTC, 1 CH) had evaluated surge capacity in connection with a disaster drill, one hospital in connection with the COVID-19 pandemic and one said that it “occurs at every specific incident”. Note that not all hospitals specified mode of surge capacity evaluation.

Regarding regional coordination, 1/4 (25%) RTCs had a plan for when and how patients will be sent to other hospitals, while this for others was 1/2 (50%) NTCUH, 10/16 (62%) CH, 8/15 (54%) CDH and 1/2 (50%) LH. 10 hospitals had no plan on how to send patients. 3 hospitals stated that they do not have disaster drills alongside the region. Among others, 4 answered "do not know”. 28 hospitals stated that exercises with the region had been carried out.

### Real-life events

Last time the contingency plan was activated for MCI was for UH in half of the cases 2–5 years ago, while a majority of CH and CDH activated the plan more than 10 years ago (17/31, 55%), but spread was large. Just over half of CDH had never activated the disaster plan for MCI.

### Supply management, mobilization at the hospital, view on key areas

In this section, many chose "cannot answer". Response base is thus limited. Of those responding, 9 CH, 11 CDH and 1 LH (21/39 in total) had no system for controlling material supply, 3/39 hospitals (1 CH, 1 CDH, 1 LH) had such system. 16 hospitals had disaster stores at the hospital, 12/39 lacked such stores (Table 3). Nine hospitals updated the disaster store annually and two less than every two years.

**Table 3:** Does your hospital keep emergency storage at the hospital in case of a disaster?

	Yes (*n*)	No (*n*)	Cannot answer (*n*)	Do not know (*n*)
RTC	2	0	2	0
NTCUH	1	0	1	0
CH	8	4	4	0
CDH	4	7	3	1
LH	1	1	0	0

*RTC* Regional trauma center, *NTCUH* Non-trauma center university hospital, *CH* County hospital, *CDH* county district hospital, *LH* local hospital. *n* 39

Among RTC, it varied greatly whether they had a system to increase capacity for patient beds or not; 1/4 (25%) had such plan, 1/4 did not, 1/4 did not know and 1/4 chose not to answer due to confidentiality. Among others, a majority had a system to increase capacity; 2/2 (100%) NTCUH, 13/16 (81%) CH, 8/15 (53%) CDH and 1/2 (50%) LH. Regarding how to distribute patients in an MI, 16/39 (41%) stated that they would be placed in the same ward and a relatively large proportion of mainly smaller hospitals that the patients should be placed in different wards (13/39, 33%; for smaller hospitals 12/31, 39%).

Respondents were asked to rate how important specific areas were considered to be for improving the hospitals disaster preparedness, on a scale of 1–10, 10 being highest. In all areas, respondents for RTC chose a lower rating compared to other hospital types (in terms of mean and median). International cooperation was ranked lowest (mean 6.47 from all respondents) followed by increased military cooperation (mean 8.35). Most important were exercise and simulation (average 9.64), and disaster management and leadership (average 9.06).

Finally, respondents were asked to openly describe what they found important for increasing hospital disaster preparedness. Three themes recurred among all hospital types; increased national collaboration and consistency of methods and contingency plans, national guidelines, and increased training in disaster medicine.

## Discussion

Emergency hospitals play a key role in society's ability to handle major incidents such as mass casualty incidents and must be prepared for such crises. Main findings of this survey study include several possible areas for improvement in disaster preparedness at Swedish hospitals. All-though response rate was lower than hoped, most of the emergency hospitals were represented (69.4%), the largest hospital in most regions participated, and almost all regions were represented. General conclusions can thereby be drawn. Legislation and guidelines were largely complied with and results pointed to major differences in how preparedness work is conducted, for example regarding contingency plans, exercises, and triage methods. A clear majority of hospitals cover most key areas described by the WHO [11], but no hospital covers everything.

Results indicated that this method could possibly be used as a quality indicator to monitor that hospitals maintain similar and adequate levels of preparedness. However, the survey was only tested once in this study and should undergo further validation and testing before clear conclusions on this can be drawn. In combination with specified requirements on what functions and levels of education should be available for each hospital type, a survey like the presented, addressed by accredited organizations or authorities, could make hospitals and regions uniformly prepared and contribute to making hospitals better equipped for MIs. If results are openly presented and carried out at regular intervals, it could function as a trigger for improvements [1, 11].

Large differences in the hospitals’ contingency plans were seen, even though this is known to worsen conditions for coordinating efforts in MIs [1]. In this study, implementation of contingency plans among health-care staff also varied. The fact that contingency plans at several hospitals was very long increases the risk of staff not reading it [1, 11]. The importance of increased coherence and collaboration is emphasized in recently completed studies on Swedish health care's disaster preparedness [14].

Over the past 20 years, the number of hospital beds in Sweden has gradually decreased to the lowest level in the OECD [15]. In the same period, occupancy rate has increased. As this is strongly linked to hospital capacity [1], trends of increased overcrowding can be seen as a sign that disaster preparedness has deteriorated. Reduced number of hospital beds and ventilators combined with a lack of material supply also constitutes three of five main limiting factors for hospitals ability to handle MCIs found in a study from 2020 [15] of Swedish surgical surge capacity, where large regional differences were seen [15]. In March 2020, the Canadian Association of Emergency Physicians (CAEP) stated, that Canadian healthcare, contrary to the opinion of many authorities, was dangerously unprepared for disasters, and active efforts were required to fill identified gaps [16]. In line with Blimark et al. [15], this study has indicated a need for increased hospital surge capacity. The trend of declining numbers of hospital beds must be reversed to create conditions for improved disaster preparedness. In combination with increasingly subspecialized and fragmented healthcare, which has a negative impact on trauma care [16–18], reduced margins could be seen as one of the biggest challenges in maintaining sufficient capacity in Swedish hospitals.

According to the Health and Medical Services Act [6], a function or group at hospital level responsible for preparedness is recommended to best mobilize resources [1, 19]. A clear majority of those answering the survey have coordinating functions, but the fact that the figure does not reach 100% leaves room for improvement. Without a function responsible, there is a risk that review of contingency plans become less frequent and less structured, exercises fewer and general interest in disaster preparedness at the hospital risks diminishing [9, 11, 12].

Specific training requirements for coordinating functions varied greatly between hospital types. In Sweden, disaster medicine is no longer part of basic medical education for future doctors and is limited to 1–2 weeks during basic education for nurses. Formal education in disaster medicine is conducted in the form of separate courses through different universities, organisations (for example the MRMI courses) and the Centre for Disaster Medicine and Traumatology in Linköping, Sweden. Smaller hospitals generally place higher demands for formal education in disaster medicine for coordinating functions compared with university hospitals, which was surprising. Larger hospitals may generally have more experience in trauma care and could consider themselves to have sufficient practical experience. However, experience from trauma in everyday life does not reflect on ability to adapt to special working methods required in an MCI [1, 15]. In addition, trauma patient volume at Scandinavian hospitals has been shown to be insufficient for maintaining competence [17]. Formal education in disaster medicine could work as a quality assurance for competence and should be a basic requirement for those responsible for the hospitals’ disaster preparedness.

Most hospitals have a multidisciplinary Hospital Command Group (HCG), but several smaller hospitals only have regional management or decide who should be included in connection with the major incident. This despite the fact that contingency plans, according to national guidelines, clearly must state who has the initial management responsibility in an MI [7] (Fig. 1). The fact that HCG has not been defined in advance poses a great risk that establishment of management and thus the entire effort is delayed. Different working methods in hospitals’ HCG can lead to difficulties with coordination between regions. Several hospitals do not require leadership training for those in the HCG, which goes against research and recommendations [1]. Improved management structure was the key area for improving preparedness ranked second highest by respondents.

Uncertainty in surge capacity risks leading to hospital overloading in an MCI. Overloading could cause normal quality requirements not being maintained despite adequate measures [1]. This affects patients through reduced quality of care despite sufficient capacity being available at other, less affected, hospitals in the immediate vicinity. Distributing patients in a disaster is a major logistical challenge, but knowledge on capacity and limit for each hospital can facilitate the task and be seen as a prerequisite. The fact that many hospitals lacked a plan for when and how patients are to be transported to different hospitals complicates the task even more. This study does not test capacity, but results indicate that capacity constraints highlighted by the NBHW [20] 4 years ago have not sufficiently been taken into account in the hospitals' disaster planning.

Regarding supply management in an MI, areas for improvement are seen at most hospitals. Many regions worked according to the "just-in-time" principle, which for instance led to lack of personal protective equipment when demand increased at the start of the COVID-19 pandemic 2020 [5]. In a sudden MI such as an MCI, there is a risk of rapid material consumption, and it is reasonable that each hospital has stockpile to manage for the first day. In addition, more hospitals should include material supply in surge capacity evaluations and contingency plans.

Triage methods used in a disaster differ greatly between hospitals and regions. Several planned on using regular triage methods, i.e. methods not adapted to disasters such as the RETTS system. The use of many different methods also poses a risk of miscommunication as patients are sent between hospitals and regions. A common triage method intended for disasters, for instance, physiological triage systems such as triage sieve and triage sort as initial triage followed by an anatomical triage, should be implemented at all hospitals in Sweden.

Swedish hospitals have been relatively spared from MCIs, making exercises essential. [1, 20]. Several hospitals rarely had drills, and two did not train at all. This highlights an obvious gap in disaster preparedness. Among RTCs, frequency of exercises was lower than expected. Contingency plans thereby risk being insufficiently tested. Exercise and simulation also create the opportunity to evaluate hospital surge capacity and has in a study [21] after the terrorist attacks in Manchester 2017, been shown valuable for staff confidence in decision-making in MIs, which is in line with several other studies on the subject [1, 8, 10, 21–25].

In an MCI, there are great risks that communication systems are overloaded [1, 19]. That a large proportion of hospitals plan on using regular systems in an MI creates an obvious risk of communication failure, which happened during the terrorist attack in Stockholm 2017 [8]. Communication failure could delay and impair care and is often an overlooked area [19]. Alternative communication systems in the event of disturbances must be in place. Backup systems for communication were also found in a study from 2020 [19] to be lacking in many trauma centres in Canada, Australia, New Zealand and the United Kingdom. Gabbe et al. [19] conducted a questionnaire study on all trauma centres in the above-mentioned countries, and their results are in line with those obtained through this study. The areas of improvement identified thus do not appear to be unique to Sweden but are found in several countries with similar healthcare systems.

The respondents ranked international and military cooperation to be of lowest value for improving hospital disaster preparedness. This may have since changed, as Sweden now experience a vastly different security situation and level of threat in the immediate area.

The study has a good geographical coverage with 18/21 regions represented, which means that trends seen can be considered to apply to the whole country. Whether the results are transferable to other countries is more uncertain as healthcare systems and guidelines differ. However, the method used could be used for control and follow-up in other contexts, as the areas explored are fundamental to all healthcare systems.

### Limitations

The study would clearly benefit from a larger response rate as issues about national representability can be raised. However, as declared above, almost all regions were represented and a good geographical spread was achieved. In addition, the response rate among emergency hospitals, which were the main target of the study, was considerably higher than among all invited hospitals. Surveys carry inherent limitations and risks for misinterpretation of questions. Results should be interpreted with care. There is a risk, that respondents wanted to give a better picture of preparedness at the hospital they represented than the actual, and pressure from hospital leaders to paint a better picture than the real is always a risk. Measures were taken to overcome this by anonymity. Many of the questions are easy to validate. In addition, answers partly reflect a perceived description of reality. As MIs are unusual, it is difficult to test how well the perceived image of hospital preparedness matches reality. To obtain this, realistic simulation exercises and surge capacity tests, are needed. [26, 27] As the survey was aimed at and mainly answered by contingency coordinators, the prerequisite for correct answers has been optimized. Future studies validating this method using this or similar surveys, are needed.

## Conclusions

Swedish hospitals' disaster preparedness generally covers many key areas, but no responding hospital seems to cover all. Several areas for improvement were found, especially within implementation and overall extent of contingency plans. Frequency of exercises differed greatly and almost half of hospitals did not perform surge capacity evaluation. University hospitals have lower requirements for formal education in disaster medicine for key functions. Large differences between hospitals identified in this study offers difficulty in achieving national equality regarding preparedness. Several local contingency coordinators requested national consistency and guidelines for contingency plans. Future in-depth studies and simulation exercises using existing resources are needed to test several of specific areas discussed. The survey used in this study was found useful as an overview of hospital disaster preparedness and should be further evaluated and conducted at regular intervals.

## Appendix

### Appendix 1 – survey

#### Questions about the hospital and its coordinating functions

**1.** Which hospital do you work at? *

**2.** What type of hospital is it? *

- Regional Trauma Centre
- Non-Trauma Centre University Hospital
- County Hospital
- County District Hospital
- Local Hospital

**3.** Approximately how many inhabitants does the hospital have in the catchment area for trauma care? *

-  > 1,000,000
- 500,000–1,000,000
- 100,000–500,000
-  < 100,000

**4.** What function do you have, that is responding on behalf of the hospital? *

- Contingency Coordinator
- Contingency Physician
- Head of trauma or emergency department or other function with comprehensive knowledge on local hospital disaster preparedness
- Other, please specify:

**5.** Does your hospital have a designated person/function responsible for disaster preparedness (e.g. contingency coordinator)? *

- Yes, the hospital has a function with time designated for disaster preparedness issues.
- Yes, the hospital has a function, however without time designated for disaster preparedness issues
- No, we do not have a designated function
- Do not know

**6.** If your hospital has a contingency coordinator, please specify staff category: *

- Nurse, specify specialty:
- Doctor, specify specialty:
- Other, please specify:
- Do not know

**7.** What type of training in disaster medicine does your contingency coordinator have? *

- No formal education in disaster medicine
- Formal education in disaster medicine, please specify what type of education and year for latest course:
- We do not have a contingency coordinator
- Do not know

**8.** Does your hospital have a contingency physician? *

- Yes
- No
- Do not know

**9.** If your hospital has a contingency physician, please specify which specialty: *

- Surgery
- Anesthesia/intensive care
- Orthopedics
- Internal medicine
- Other, please specify:
- We do not have a contingency physician
- Do not know

**10.** What type of training in disaster medicine does your contingency physician have? *

- No formal education in disaster medicine
- Formal education in disaster medicine, please specify what type of education and year for latest course:
- We do not have a contingency coordinator
- Do not know

**11.** Does your hospital have a disaster committee (or similar)? *

- Yes
- No
- Do not know

**12.** If your hospital has a disaster committee, please indicate which functions are represented in the committee:

- Secretary
- Administrative staff
- Chief function
- Surgery
- Orthopaedics
- Anaesthesia and intensive care
- Nursing staff
- Security
- Psychological crisis team
- Communication and information
- Other, please specify:

**13.** Do you require a formal education in disaster medicine for those included in the disaster committee? *

- No formal education is required
- Formal education is required, please specify what type of education and year for latest course:
- We do not have a disaster committee
- Do not know

**14.** Indicate approximately how many people in your hospital have formal training in disaster medicine: *

-  > 10
- 5–10
-  < 5
- None that I know of
- Do not know

#### Questions about the contingency plan

**15.** How extensive is your contingency plan? (Several answers can be marked) *

- We have a comprehensive contingency plan for the entire hospital
- Several units have their own contingency plan, and they are synchronized with each other
- Several units have their own contingency plan, but they are not synchronized with each other
- Do not know

**16.** How many pages does your hospital-wide contingency plan contain? (Numbers) *

**17.** Briefly describe how your contingency plan is organized.

**18.** When was your contingency plan last updated? *

- Less than one year ago
- More than one year ago
- 2–3 years ago
-  > 3 years ago
- Do not know

**19.** When was the section for mass casualty incidents last updated? (Year) *

**20.** Have all parts of the contingency plan been updated? *

- Yes
- No
- Do not know

**21.** How often is your contingency plan practiced? *

- More than once a year
- Every year
- Every two years
- Less than every two years
- It is not practiced
- Do not know

**22.** Who reads your contingency plan? (Choose the option that is most accurate) *

- All new staff reads it upon employment
- All staff according to a guideline
- It is up to each individual to read it according to their own needs
- Other, please specify:
- Do not know

**23.** What scenarios does your contingency plan contain? (Tick all matching options) *

- Mass casualty incidents
- Fire
- CBRN (Chemical, Biological, Radiological, Nuclear)
- Epidemic
- Hypothermia (drowning)
- Inhalation injuries
- Security threats towards the hospital
- Natural disasters
- Psychological crisis support/psychological trauma
- Evacuation of the hospital
- Network/IT disruptions
- Other, please specify:

#### Questions about action cards

**24.** Which functions within the hospital have action cards? (Tick all suitable) *

- Doctor in the emergency room
- Surgeon in the emergency room
- Surgeon in the surgical ward
- Triage doctor
- Nurse in the emergency room
- Nurse in the surgical ward
- Nurse in the ICU
- Nurse in the postoperative ward
- Radiologist
- Nurse in radiology
- Anaesthetist in the emergency room
- Anaesthetist in the surgical ward
- Anaesthetist in the ICU
- Anaesthetist in the postoperative ward
- Doctor in medical ward
- Nurse in medical ward
- Doctor in outpatient clinic
- Nurse in outpatient clinic
- Blood centre
- Lab
- Health technology
- Patient transport
- Secretary
- Telephone operators
- Hospital security
- Psychological crisis support team
- Hospital Command Group
- Other, please specify:
- Do not know

#### Questions about disaster drills

**25.** Which employees are involved in disaster drills? (Choose the option that is most accurate)*

- All employees receiving trauma patients
- We train a few individuals every year according to a rotating schedule
- Most often, those who are most interested take part in the drill
- We do not have disaster drills
- Other, please specify:
- Do not know

**26.** Do you practice different scenarios in your disaster drills? *

- Yes, we vary between traumas and other scenarios such as gas leak, fire etc.
- Yes, but only different types of physical trauma
- No, we don’t practice for different scenarios
- No, we do not have disaster drills
- Other, please specify
- Do not know

**27.** How do you evaluate your disaster drills? (Choose the option that is most accurate) *

- Structured review within the disaster committee
- Surveys (or similar) before and after the drill in order to evaluate if the staff has gained improved knowledge in hospital disaster preparedness and the contingency plan
- We do not evaluate our drills
- We do not have disaster drills
- Other, please specify:
- Do not know

**28.** If you evaluate your disaster drills—what are the results used for? (Choose the option that is most accurate) *

- To measure the quality of the exercise
- They are used to evaluate and improve the contingency plan
- They are used to map staff competence and optimize hospital capacity
- We do not have disaster drills
- We do not evaluate disaster drills
- Other, please specify:
- Do not know

#### Questions about Hospital Command Group (HCG)

**29.** What does your Hospital Command Group (HCG) look like in the event of a serious incident/disaster? *

- We have a trained Hospital Command Group consisting of trained people with a multidisciplinary composition
- We have a disaster management team that consists of our regular managers
- Do not know
- We have no designated disaster management/HCG
- Other, please specify:

**30.** What functions are represented in your HCG?

- Administrative staff
- Anaesthesia and intensive care
- HR
- Surgery
- Controller
- Media communicator
- Secretary
- Hospital management
- Hospital security
- Nursing staff
- Telephone operators
- Other, please specify:
- Do not know

**31.** Do you work according to a certain method of working within HCG (for example the NATO model)? *

- Yes, please specify:
- No
- Do not know

**32.** Does your HCG train for serious incidents and disasters? *

- Yes
- No
- Do not know

**33.** If your HCG trains, when did they last train for mass casualty incidents? (Year) *

**34.** Is a formal training in staff methodology required to participate in your HCG? *

- Yes
- No
- Do not know

#### Questions regarding communication and information

**35.** What type of means of communication is intended to be used in the event of a disaster? *

- Regular communication with hospital phones and pagers
- Mobile phones and landlines
- Special back-up system that has been tested for the event that normal means of communication are unavailable
- We do not have a back-up system for communication
- Other, please specify:
- Do not know

**36.** Do you have a system to ensure that there are always updated staff contact lists? (Choose the option you find most accurate) *

- Yes, specific person appointed to be responsible for updating the list
- We have updated staff contact lists without a specific system for updating
- Other, please specify:
- Do not know

**37.** How are staff informed about level of alert/ongoing serious events during regular hours? (Several options can be marked). *

- Announcement thorough speaker system in the hospital
- Telephone operators call each ward/clinic
- Text message
- Other, please specify:
- Do not know

**38.** How are staff informed about level of alert/ongoing serious events during on-call hours? (Several options can be marked). *

- Announcement thorough speaker system in the hospital
- Telephone operators call each ward/clinic
- Text message
- Other, please specify:
- Do not know

**39.** Does your hospital have an internal speaker system for notifying all staff about, for example, threats and alert levels? *

- Yes
- No
- Do not know

#### Questions about triage

**40.** Where will primary triage take place in the event of a disaster or other serious incident? *

- In the ambulance hall
- In the emergency department
- Other specified location at the hospital, please specify:
- We do not have a system for where primary triage will take place
- Do not know

**41.** Where will secondary triage take place in the event of a disaster or other serious incident?*

- In the trauma room
- We let each trauma team decide
- Other, please specify:
- Do not know

**42.** Which triage system will be used for primary triage in the event of a mass casualty incident? *

**43.** Which triage system will be used for secondary triage in the event of a mass casualty incident? *

#### Questions regarding surge capacity

**44.** Do you have an evaluation of the hospitals surge capacity? *

- Yes
- No
- Do not know

**45.** What units are included in your surge capacity evaluation? *

- Emergency department
- Intensive care
- Health technology
- Material supply
- Surgical department
- Staff resources
- Postoperative ward
- Radiology
- Medical wards
- Sterile goods unit?
- Other, please specify:
- We do not have a surge capacity evaluation
- Do not know

**46.** Describe how your surge capacity evaluation has been done

- Description:
- We do not have a surge capacity evaluation
- Do not know

**47.** When did you last evaluate the hospitals surge capacity?

- Year:
- We do not have a surge capacity evaluation
- Do not know

#### Questions about regional collaboration

**48.** Do you have a plan for how and when patients will be transferred to other hospitals within the region or in another region? *

- Yes
- No
- Do not know

**49.** Do you conduct disaster drills and collaboration with regional management? *

- Yes, we have a collaboration and train together
- No, we do not have an established collaboration and do not train together
- Other, please specify:
- Do not know

#### Questions about real life events and supply management

**50.** Has your hospital activated the contingency plan for serious incidents with many injured after physical trauma (MCI)? (Several choices possible) *

- Yes, within the last 2 years
- Yes, within the last 2–5 years
- Yes, within the last 5–10 years
- Yes, more than 10 years ago
- No, never
- Do not know

**51.** If you answered yes on question 50, did you evaluate the hospitals effort in connection with the event?

- Yes, please describe how:
- No
- Do not know

**52.** If you answered yes on question 51, did the evaluation lead to any changes in your contingency plan? *

- Yes, the contingency plan was updated based on the deficiencies identified
- No, the contingency plan was activated and worked according to plan, kept in the same form
- No, revision of the contingency plan was not included in the evaluation after the event
- Do not know

**53.** Do you have a system that has been tested and adapted for a disaster situation to keep track of material supply, sterile instruments, medicines, etc.? *

- Yes, please describe:
- No
- Cannot answer
- Do not know

**54.** Is there a material stockpile at the hospital for the event of a serious incident or disaster? *

- Yes
- No
- Cannot answer
- Do not know

**55.** How often is the hospital disaster stockpile updated?

- Every year
- Every two years
- Less than every two years
- Cannot answer
- Do not know

**56.** Are there systems for how to increase the hospital's capacity for inpatients?

- Yes, please describe: *
- No
- Do not know

**57.** How should patients be distributed in the hospital in the event of a large influx of patients, for example during an MCI? *

- Different wards
- Same ward as far as possible
- Other, please describe:
- Do not know

#### Rate how important different areas below are to improve and develop disaster preparedness at your hospital/within your region

**58.** Rate how important do you think **exercise and simulation** are to improve disaster preparedness at your hospital/within your region?

**59.** Rate how important do you think **improved disaster stockpiles** are to improve disaster preparedness at your hospital/within your region?

**60.** Rate how important do you think **improved communication systems** are to improve disaster preparedness at your hospital/within your region?

**61.** Rate how important do you think **improved/clearer management** is to improve disaster preparedness at your hospital/within your region?

**62.** Rate how important do you think **increased collaboration between different hospitals and prehospital actors** is to improve disaster preparedness at your hospital/within your region?

**63.** Rate how important do you think **increased civil and military cooperation** is to improve disaster preparedness at your hospital/within your region?

**64.** Rate how important do you think **increased international exchange/collaboration with other hospitals** is to improve disaster preparedness at your hospital/within your region?

**65.** Rate how important do you **think research and development in disaster medicine and disaster preparedness** are to improve disaster preparedness at your hospital/within your region?

**66.** Are there any other things that are important for improving the hospital's disaster preparedness? (Describe briefly).

**67.** Is there anything you would like to add?
